# Birth of a Child Without Rupture of Membranes

**Published:** 1895-08

**Authors:** 


BIRTH OF A CHILD WITHOUT RUPTURE OF MEMBRANES.—Forman (*Jour. de Med. de Paris*) observed this rare occurrence in the case of a woman, aged twenty-two, seven months advanced in her second pregnancy. She was suffering from pleuro-pneumonia, the temperature having risen to 103.6°. A few minutes after cupping glasses had been applied to the bases of the lungs the patient felt a desire to defecate; this was followed by a single pain which expelled the entire ovum with a little blood. Forman arrived a few minutes later. He found between the patient's thighs a big cyst with transparent walls. The mother was free from all the evils which may follow precipitate delivery; the uterus contracted well. The wall of the cyst was then cut; about a pint of amniotic fluid escaped. A female child was seen; there was no pulsation of the cord, but after active measures the infant breathed well and took the breast. It weighed three pounds and six ounces, and measured over fourteen inches; the placenta weighed a little under a pound. The cord was very gelatinous and measured 18½ inches in length. Judging from the position in which the ovum lay—outside the vulva—it seemed that the breech had presented, and that the placenta had been inserted very low down without being previa. The child lived only sixteen hours, and the mother had a bad attack of empyema. Forman quotes a considerable number of cases of membranes unruptured at birth. —*British Medical Journal.*

